# Evaluating the burden of caregivers of patients with visual impairment: a multicenter pilot study in Italian visual rehabilitation centers

**DOI:** 10.3389/fpubh.2025.1530172

**Published:** 2025-06-06

**Authors:** Gianni Virgili, Francesco Ferron, Federico Bartolomei, Ruth Van Nispen, Eliana Costanzo, Giovanni L. Ciaffoni, Emanuela Rellini, Simona Turco, Marina Piepoli, Simona Di Pietro, Stefania Fortini

**Affiliations:** ^1^Department of Neurofarba, University of Florence, Florence, Italy; ^2^IRCCS – Fondazione Bietti, Rome, Italy; ^3^Istituto dei Ciechi F. Cavazza, Bologna, Italy; ^4^Department of Ophthalmology, Amsterdam Public Health Research Institute, Amsterdam UMC, Vrije Universiteit, Amsterdam, Netherlands; ^5^National Centre of Services and Research for the Prevention of Blindness and Rehabilitation of Low Vision Patients, IAPB Italia Onlus, Rome, Italy; ^6^Ophthalmology Unit, Fondazione Policlinico Universitario A. Gemelli IRCCS, Rome, Italy; ^7^Clinical Psychology Unit, Fondazione Policlinico Universitario Agostino Gemelli IRCCS, Rome, Italy; ^8^Eye Clinic, Department of Medical Science, Neuroscience and Sense Organs, University of Bari, Bari, Italy; ^9^Azienda Ospedaliera Policlinico Tor Vergata, Rome, Italy

**Keywords:** caregiver burden, vision impairment, vision rehabilitation, Italian low vision, CBI, IADL

## Abstract

**Introduction:**

Despite growing evidence that underscores the importance of the caregiver’s role in the rehabilitation process, visual rehabilitation (VR) programs often overlook these needs. The aim of this pilot study is to investigate the caregiving burden (CB) among informal caregivers of visually impaired (VI) patients who attend Italian VR centers, setting the bases for large-scale research.

**Methods:**

Four Italian VR centers were involved. Demographic data and IADL (Instrumental Activities of Daily Living Scale) questionnaire to assess the degree of autonomy of VI patients were collected. Regarding the caregiver, the Italian validated version of the “Caregiver Burden Inventory (CBI)” was administered.

**Results:**

Fifty patients and their caregivers were included. The mean total CBI score was 23.6 points (SD 18.4), which is about the threshold for abnormal stress score. Moreover, 9 (18%) caregivers had scores ≥39, suggesting burn-out or mental disorder. The time-dependent (rho = 0.88), developmental (rho = 0.93), and physical burden (rho = 0.87) domains demonstrated the strongest correlations with overall CBI score. Furthermore, each additional hour of caring increased the score by 1.07 points (*p* = 0.004). No association was detected between total CBI score and other patient’s characteristics, including dual sensory deficit (auditory and visual), as well as patient’s IADL score.

**Conclusion:**

In this pilot study in VI patients attending VR services, about one half of caregivers of VI patients experienced stress, with 1 in 5–6 suffering from burn-out or mental health issues. Larger studies should assess both the outcome and the resources needed to screen for CB, with care integrated in the patient rehabilitation pathway.

## Introduction

In a global society built on the ability to see, visual impairment (VI) has far-reaching consequences for individuals, their families and caregivers (1), many of which can be mitigated by timely access to quality eye care and rehabilitation (2). The term “caregiver” is generally defined as “someone who provides care for a person who is unable to care for themselves.” (3) In the Italian legal system, the figure of the “family caregiver” is the persons who organizes and defines the care needed by another person, often a relative, and is generally a primary family member (4). Family caregivers play a central role in the life of the sick person, both in day-to-day care and as the emotional reference for the person being cared for. The main difference between a family caregiver and a professional caregiver is therefore the unpaid, voluntary nature of the service.

A large survey on health and health service use in Italy and the European Union in 2015 estimated the number of caregivers in Italy to be between 7 and 12 million (5). In another survey focusing on older adult people, conducted in the same year, caregivers were mostly women aged between 45 and 64 (6). In fact, almost 30% of women aged 45–54 provided care.

The care provided by a family or informal caregiver is essentially a job, often undertaken out of love for a family member, rather than by choice. Moreover, informal caregivers often lack the necessary rest, vacations, sick days, or time for medical check-ups and preventive screenings, which a professional caregiver or home carer (formal caregiver) is entitled to (4–7).

When the burden of care becomes overwhelming, caregivers may be at greater risk for mental and physical health problems (8). This is more likely to occur when the caregiver has difficulty balancing their own needs with those of the family member, or when family financial resources are constrained (9, 10).

Extensive research on CB for caregivers of people with VI has been conducted in clinical settings, where patients with exudative AMD receive intravitreal injections (9–13). These studies have shown multidimensional caregiver overload, with a profile similar to that in other clinical conditions. However, research on CB conducted in VR settings, which help patients use residual resources and cope with their disability, is scarce.

Thus, we planned this pilot study to investigate the CB in patients attending VR services in Italy, exploring its correlation with routinely collected caregiver’s and patient’s characteristics, with the goal of supporting the design of larger registry-based multicenter studies.

## Materials and methods

### Study design and included patients

This is a multicenter observational pilot study conducted in Italy, involving four Italian VR centers (Bari, Florence, Rome “Tor Vergata,” Rome “Gemelli”). The study population consisted of patients aged 18 or older, affected by any visual disease, attending these VR specialized centers. We excluded patients with cognitive impairment who are unable to understand informed consent or complete questionnaires. All centers enrolled patients between January and March 2024. They usually included one patient per session, i.e., the first caregiver-patient dyad who agreed to provide data for the study, without specific restrictions concerning the caregiver relationship with the VI patient. The goal was to obtain a heterogeneous sample of patient-caregiver dyads, in order to refine the data collection and the general methodology to be adopted in larger registry-based studies.

Data obtained from low-vision patients attending the VR centers involved in the study are routinely stored in a web register (D.A.Re. INVAT) that was developed to track the patient’s profile and rehabilitation process as a tool for both research and management (14–16). D.A.Re. records demographic, clinical and functional data, together with visual aids in use and prescribed. Additionally, one validated questionnaire is always administered to the patient: the IADL (Instrumental Activities of Daily Living Scale) to assess the degree of independence in instrumental activities (ability to use phone, shopping, food preparation, housekeeping, laundry, mode of transportation, responsibility for own medications, ability to handle finances), with score range 0–8, where 0 express dependence and 8 indicates complete independence.

### Caregiver burden assessment

Caregiver burden was assessed using the 24-item, five-subscale Caregiver Burden Inventory (CBI) which was developed (Supplementary material) for use in professional and family caregivers (17–19). This self-report tool was developed for caregivers of patients with Alzheimer’s disease and related dementias. The CBI is divided into five sections dealing with different factors of stress: time-related load, psychological load, physical load, social load, emotional load (17, 18). In a very recent work, the CBI was listed as one of the best self-assessment instruments for informal caregivers (18). The CBI has the added benefit of including an assessment of developmental burden, which informs on the long-term consequences of caregiving when it disrupts the life course development of caregivers (19). The CBI is a quick and easy-to-understand tool, which can be self-administered. Divided into 5 sections, its domains are defined as follows:

- Time dependence: burden dependent on the time required for care, or the burden associated with time restrictions for the caregiver;
- Developmental burden: the evolving burden, understood as the caregiver’s perception of feeling cut off from the expectations and opportunities of their peers;
- Physical burden: feelings of chronic fatigue and somatic health problems;
- Social burden: which describes the perception of a role conflict;
- Emotional burden: which describes feelings towards the patient, which may be induced by unpredictable and bizarre behaviors.

A score at 0 means the caregiver thinks the item is “not at all” (then no burden), and a score at 4 means “very much so” (very relevant burden). An overall score of 24 was considered a threshold for unhealthy values, and a score of 39 or more suggested burn-out or mental disease (19, 20). For caregivers, data was also collected on: age, gender, degree of kinship with the care recipient, and number of daily hours spent providing care. Caregiver data was anonymized and associated with their care recipient through the patient’s ID in the D.A.Re. register, which can only be decoded at the center where the patient is examined.

The study design was approved by the Ethics Committee of the Tuscany Region (Comitato Etico Regionale per la Sperimentazione Clinica della Regione Toscana: code 15992).

### Statistical analyses

Statistical analyses were largely descriptive, given the relatively small sample size and the complexity of this field. We used Spearman correlation coefficients to test the association between continuous variables in a non-parametric manner in order to avoid biases related to outlying or influential observations. No adjustment for multiple testing was adopted due to the exploratory nature of this study, as it is mainly a pilot for preparing further large-scale research. The total raw score was calculated by summing the points assigned to each of the 24 items.

Stata 19.5 software (StataCorp, College Station, TX) was used for analyses.

## Results

### Characteristics of included caregivers and patients

We included 50 patients with their respective caregivers. Caregivers were mostly female (n.30), with mean age 53.9 years (SD: 5.5 years; range: 42–72 years). The majority of caregivers were patients’ children (n.32), followed by partners (n.8), parents (n.3), carers (n.2), and other types of caregivers (n.4).

Patients’ mean age was 72.4 years (SD: 17.1 years). Regarding average visual function, better-eye visual acuity (BCVA) was 0.83 logMAR (SD: 0.60 logMAR), and reading speed was 64.6 words/min (SD: 52.3 wpm). The average IADL score was 5.2 (SD: 1.3). Three quarters of the patients were retired (n.38), 10% were employed or students (n.5), and the others were looking for a job, not seeking one, or unable to have one because of vision problems. The most common ocular condition was age related macular degeneration (AMD) in 34 patients (68%), followed by retinal dystrophy (7, 14%), diabetic retinopathy, (6, 12%) and other diagnoses.

Consistent with our previous findings (14–16) there was a long lapse between the reported onset of VI and access to our VR services, since 20 patients (40%) reported a latency of more than 4 years, and only 16 (32%) of less than 2 years. Nonetheless, as many as 27 patients (54%) already owned one or more VR devices, meaning that they had seen professionals who can prescribe VR devices.

### Overall and domain-specific CBI score

**Figure 1:**
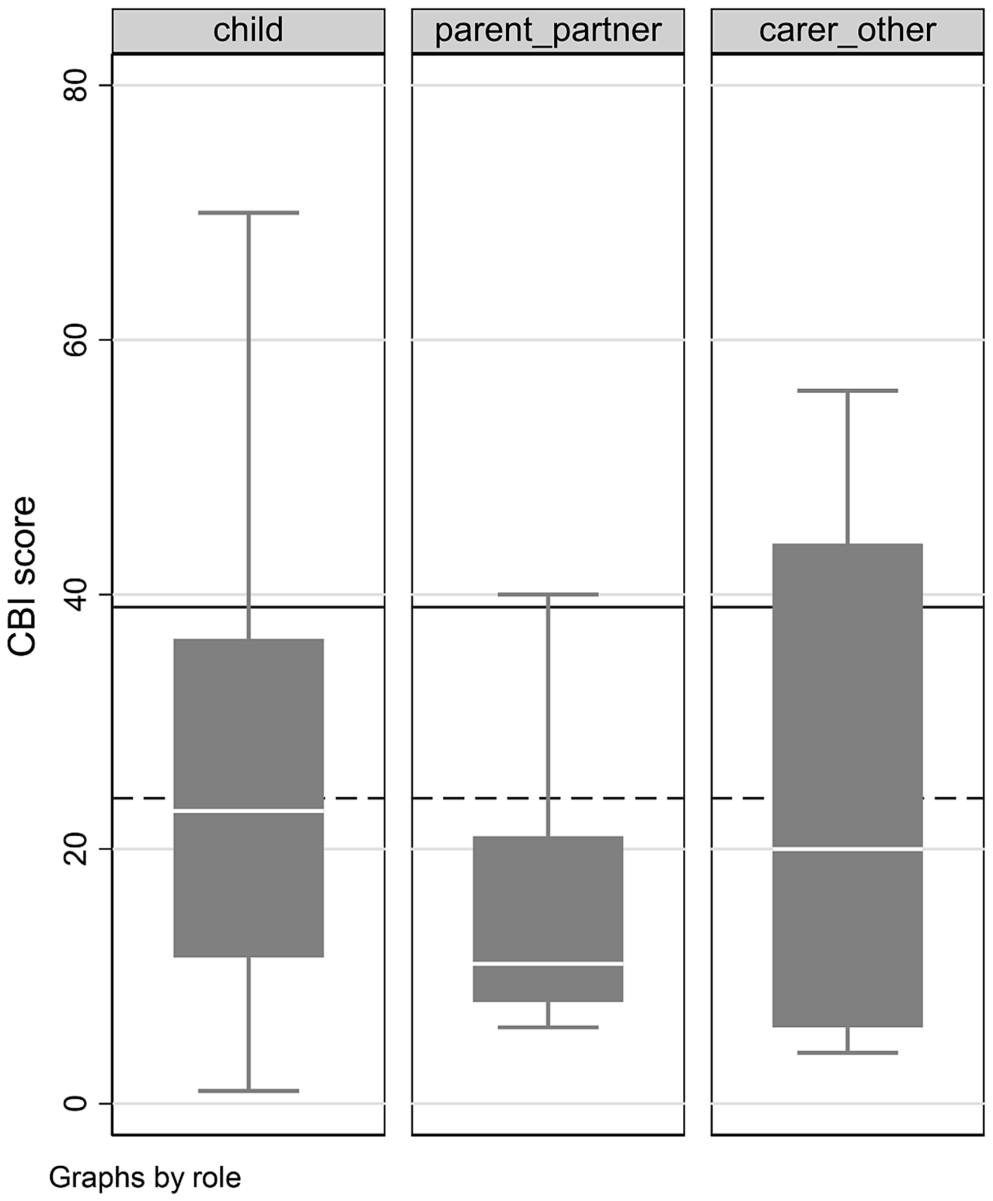
Boxplots presenting the median caregiver burden inventory (CBI) score (central horizontal line in the box), interquartile interval (box), range (whiskers) and outliers (individual dots) for different caregiver subgroup.

The mean overall CBI score was 23.6 points (SD: 18.4, range 1–70), indicating a substantial level of burden, as well as substantial score variability. Specifically, an abnormal overall CBI score (24+) was found in 18 (36%) caregivers, with a mental health disorder or burn-out (39+) in 9 (18%) caregivers. Figure 1 shows the large variability in overall score within caregiver categories, particularly children, with a sizeable fraction being above the threshold for abnormal results (lower dashed line, score: 24) and some reaching the threshold for burn-out or mental disorder (upper solid line, score: 39). Both the small sample size and the large variability made it difficult to identify differences among caregiver roles despite a 10-point lower average score for partner and parents vs. children, and 16 of 32 children (50%) vs. 9 of 11 partners and parents (82%) above score 24.

**Figure 2:**
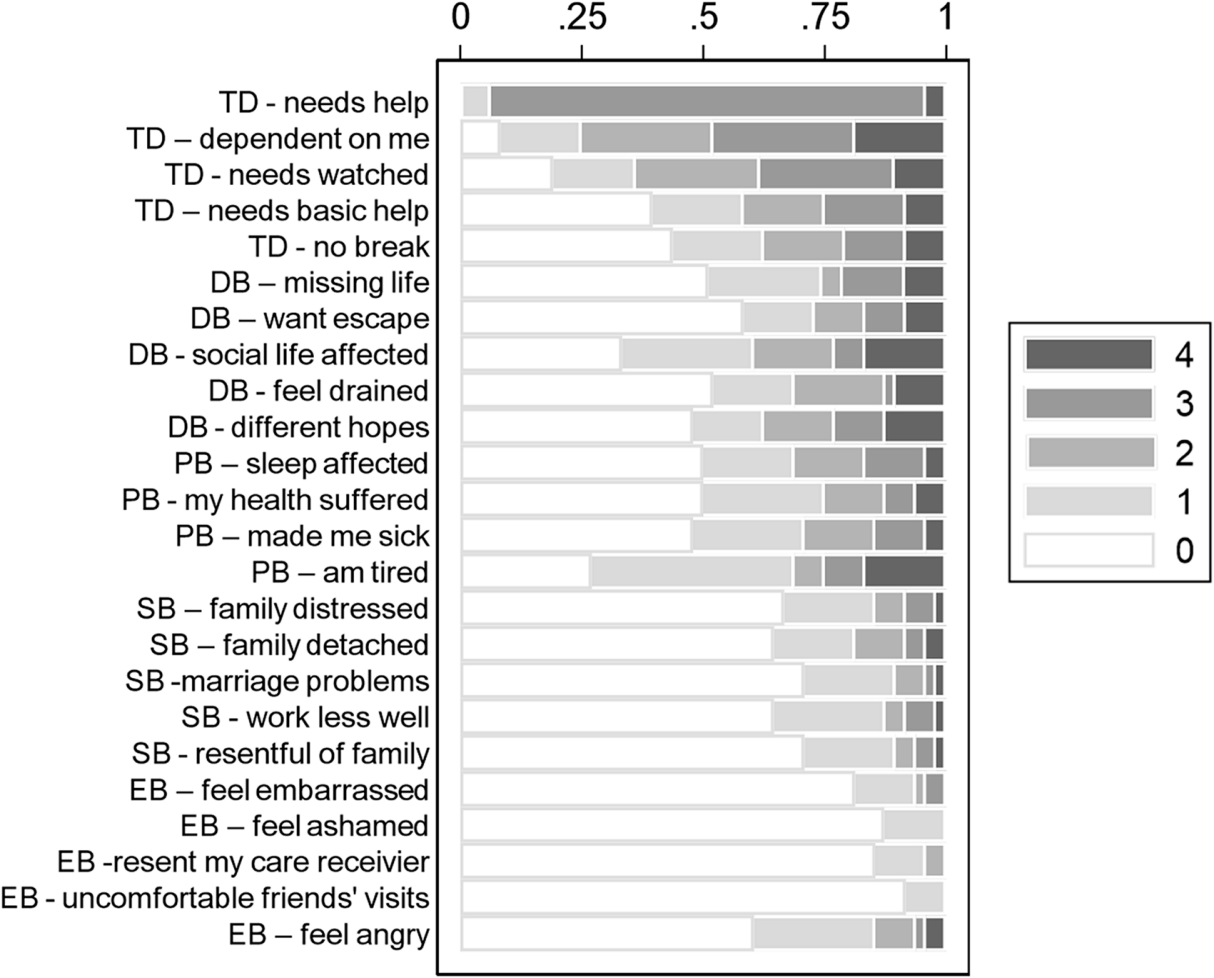
Raw scoring categories for each caregiver burden inventory (CBI) score (distribution of the responses for each item). Lower scores mean a greater need of patient help from the caregiver. The white color “score 0” means no burden at all, and, at the other extreme, the black one “score 4” means a heavy burden. CBI domains are shown as an initial two-letter acronym for each item: TD, time dependent burden; DB, developmental burden; PB, physical burden; SB, social burden; EB, emotional burden.

Figure 2 shows the distribution of the responses for each item. In this figure, the white color “score 0” means no burden at all, and, at the other extreme, the black one “score 4” means a heavy burden. As an example, regarding the time-dependent burden “needs watched,” the caregivers are spread almost equally across all scores. The *time-dependent domain*, i.e., the burden associated with personal time restrictions due to caregiving workload (mean = 1.95, SD = 0.95), and the *developmental domain*, i.e., the caregiver perception of being excluded from expectations and opportunities of same-age people, (mean = 1.14, SD = 1.22) were the most significant contributors to the overall CBI score. The *physical domain*, i.e., the feeling of chronic fatigue and somatic symptoms (mean = 1.10, SD = 1.16), also yielded high scores. Instead, low burden scores were observed for the *social domain*, i.e., the role conflict in their family environment due to caregiving (mean = 0.55, SD = 0.71), and the *emotional domain*, the feeling towards the assisted patient (mean: 0.27, SD = 0.44).

Consistent with the observed item responses, the strongest correlations with the overall CBI score were recorded for the developmental burden domain (rho = 0.93), the time-dependent burden (rho = 0.88), and the physical burden (rho = 0.87). The Spearman correlations ‘between CBI domains varied between 0.35 and 0.84, suggesting good consistency, the strongest correlation found between the developmental and physical burden domains (Table 1).

**Table 1:** CBI score correlation (Spearman rho) with caregiver characteristics and individual CBI domains (lower-left triangle) and patients’ characteristics (upper-right triangle).

		CBI score	IADL score	Patient age	Patient sex	Visual acuity (better eye)	Contrast sensitivity (better eye)	Reading speed (better eye)	CBI vs patient’s characteristics
Hours worked/day	0,36*	1	0,14	0,10	−0,34*	−0,02	−0,27	−0,01	CBI score (caregiver)
Caregiver age	−0,07	0,01	1	0,04	0,08	−0.02	−0.41	−0.01	IADL score (patient)
Caregiver sex	−0.09	−0.26	−0.15	1	−0,18	0.27	−0.30	−0.11	Patient age
Time-dependent	0,86**	0,51**	0,03	−0.16	1	−0.42*	−0.02	0.03	Patient sex
Developmental domain	0,92**	0,27	−0,08	−0.12	0,74**	1	0.02	0.20	Visual acuity (better eye)
Physical domain	0,87**	0,24	−0,09	−0.10	0,65*	0,65*	1	−0.26	Contrast sensitivity (better eye)
Social domain	0,63**	0,02	−0,13	−0.08	0,36*	0,36*	0,49*	1	Reading speed (better eye)
Emotional domain	0,67**	0,12	−0,05	−0.01	0,47*	0,47*	0,58*	0,65**	
CBI vs caregivers’ characteristics	CBI total score	Hours worked/day	Caregiver age	Caregiver sex	Time-dependent domain	Developmental domain	Physical domain	Social domain	

### Association of caregiver’s characteristics with CBI score

Caregiver’s age (Spearman rho = −0.070, *p* = 0.805) and sex (rho = −0.09, *p* = 0.345) were not associated with the total score. Instead, the number of caregiving hours per day showed a moderate association with overall score (rho = 0.36, *p* = 0.017). Although parents and partners seemed to have a lower CBI score by about 10 points (as seen in Figure 1), the estimates largely overlapped with those of children and other carers. *Association of patient’s characteristics with CBI score.*

There was no association between caregiver total CBI score and patient IADL score (rho = 0.14, *p* = 0.975). The CBI score was not associated with patient age (rho = −0.07, *p* = 0.534), but a borderline association was found with patient sex (rho = −0.34, *p* = 0.035, confirmed with a Wilcoxon test) with median score 26 for females and 11 for males. No significant correlation could be detected between CBI score and patient’s better-eye VA and maximum reading speed, and, in a subset of 15 patients, contrast sensitivity.

In a subset of 40 patients with data on hearing loss, 15 reported to be affected (37.5%), and we did not record a significant association between the presence of dual sensory loss and CBI score (rho = 0.07, *p* = 0.063, confirmed with a Wilcoxon test).

## Discussion

We conducted this pilot study to investigate the need for rehabilitation and support not only for the VI persons attending VR services, but also for their caregivers. The analysis of the caregivers’ health status shows a critical picture in many cases, which could negatively impact the care pathway of the VI patients themselves. Consistent with our findings, previous studies on CB, which were conducted mainly on caregivers of patients receiving intravitreal injections for AMD have shown a similar multidimensional caregiver overload (9–13).

Our results agree with previous studies highlighting the weight of time-related burden. Similarly, Kuriakose et al. (12), in their systematic review reported that increased number of hours of supervision to the patients play a role in caregiver’s depression and burden. The strong correlation (rho = 0.93) we found between the total burden and the developmental burden means that the more a caregiver feels “cut off” from their social life, the higher their overall burden score. Social isolation may therefore exacerbate the caregiver’s stress, and adequate social support could potentially mitigate this situation.

The physical burden showed a moderate-strong correlation with the developmental and time domains (rho = 0.65). While the relationship is less explicit in this case, this suggests that the physical fatigue associated with caregiving may limit the caregiver’s energy and availability for social activities, intensifying feelings of isolation. It is widely demonstrated that the presence of a dual sensory deficit increases the risk of developing dementia, falls, and social isolation (21). The correlation of dual sensory impairment with CB deserves to be evaluated in future studies, including qualitative research, though we did not record a significant association with CB in a subset of patients in our study.

A systematic review of studies including VI patients revealed a strong association between CB and depression (12). Factors such as a high caregiving workload, comorbidities in either the patient or caregiver, and non-adherence to visual rehabilitation programs were significantly correlated with the development of depressive symptoms (12). Intervening with individualized psychological support could be crucial to prevent the caregiver’s psycho-emotional condition from worsening (9, 10, 12). Acknowledging the underestimated impact of CB, treatments are being investigated to support caregivers of VI patients. Jin et al. (22), randomized 96 informal caregivers to cognitive behavioral therapy for 10 weeks or waiting list, with the option to participate in group treatment, also by telephone. Clinically relevant improvements were reported on depression, caregiver burden and other outcomes, though these did not each statistical significance. Moreover, approximately one third had stopped treatment, the most common reasons being lack of time and intensity of treatment.

Modern VR has progressed towards a broad, multidisciplinary approach, where mental health is a central issue, given the high frequency in VI people (23). The studies reported here remark the importance of CB in VI patients’ family care, and provide a framework for further, contextualized investigations.

The main limitation of our study is the small sample size of patient-caregiver dyads. Another limitation was that we applied no restriction to the caregiver relationship with the VI person as inclusion criterion without exclusion of professional or paid caregivers, since we wanted to collect an unselected sample, though a small one. Nonetheless, only two dyads included paid caregivers.

In conclusion, this pilot study has investigated the spectrum of CBI score in patients attending Italian VR services. The results confirm the need for further research aiming to investigate CB and the need for caregiver’s mental health support in the VR process and test whether this approach could achieve better rehabilitation outcomes for both dyad members (24).

We aim to incorporating the CBI, together with relevant caregiver’s characteristics, in the D.A.Re. registry, which collects standardized data from VR services in several Italian regions.

## Data Availability

The original contributions presented in the study are included in the article/Supplementary material, further inquiries can be directed to the corresponding author/s.
