# Supplementary material for: Evaluating the burden of caregivers of patients with visual impairment: a multicenter pilot study in Italian visual rehabilitation centers
Source: Front Public Health. 2025 Jun 6;13:1530172. doi: 10.3389/fpubh.2025.1530172 (PMC12178843; doi:10.3389/fpubh.2025.1530172)
Supplement: Supplementary file 1 [file Table_1.docx]

Appendix

1. CAREGIVER BURDEN INVENTORY (CBI) (Italian validated version)

*(Novak M. e Guest C., Gerontologist, 29, 798-803, 1989)*

0= per nulla 1= un poco 2= moderatamente 3= parecchio 4=molto

| T-D1. Il mio familiare necessita del mio aiuto per svolgere molte delle abituali attività quotidiane | 0 1 2 3 4 |
| --- | --- |
| T-D2. Il mio familiare è dipendente da me | 0 1 2 3 4 |
| T-D3. Devo vigilarlo costantemente | 0 1 2 3 4 |
| T-D4. Devo assisterlo anche per molte delle più semplici attività  quotidiane (vestirlo, lavarlo, uso dei servizi igienici) | 0 1 2 3 4 |
| T-D5. Non riesco ad avere un minuto di libertà dai miei compiti di assistenza | 0 1 2 3 4 |
| S6. Sento che mi sto perdendo vita | 0 1 2 3 4 |
| S7. Desidererei poter fuggire da questa situazione | 0 1 2 3 4 |
| S8. La mia vita sociale ne ha risentito | 0 1 2 3 4 |
| S9. Mi sento emotivamente svuotato a causa del mio ruolo di assistente | 0 1 2 3 4 |
| S10. Mi sarei aspettato qualcosa di diverso a questo punto della mia vita | 0 1 2 3 4 |
| F11. Non riesco a dormire a sufficienza | 0 1 2 3 4 |
| F12. La mia salute ne ha risentito | 0 1 2 3 4 |
| F13. Il compito di assisterlo mi ha resa più fragile di salute | 0 1 2 3 4 |
| F14. Sono fisicamente stanca | 0 1 2 3 4 |
| D15. Non vado d’accordo con gli altri membri della famiglia  come di consueto | 0 1 2 3 4 |
| D16. I miei sforzi non sono considerati dagli altri familiari | 0 1 2 3 4 |
| D17. Ho avuto problemi con il coniuge | 0 1 2 3 4 |
| D18. Sul lavoro non rendo come di consueto | 0 1 2 3 4 |
| D19. Provo risentimento verso dei miei familiari che potrebbero  darmi una mano ma non lo fanno | 0 1 2 3 4 |
| E20. Mi sento in imbarazzo a causa del comportamento del mio  Familiare | 0 1 2 3 4 |
| E21. Mi vergogno di lui/lei | 0 1 2 3 4 |
| E22. Provo del risentimento nei suoi confronti | 0 1 2 3 4 |
| E23. Non mi sento a mio agio quando ho amici a casa | 0 1 2 3 4 |
| E24. Mi arrabbio per le mie reazioni nei suoi riguardi | 0 1 2 3 4 |

1. ATTIVITA' STRUMENTALI DELLA VITA QUOTIDIANA (IADL) (Italian validated version)

*(Lawton M.P. e Brody E.M., Gerontologist, 9:179-186, 1969)*

Capacita' di usare il telefono:

| 1 | Usa il telefono di propria iniziativa |
| --- | --- |
| 1 | Compone solo alcuni numeri ben conosciuti |
| 1 | Risponde ma non è capace di comporre il numero |
| 0 | Non risponde al telefono |
| NA | Non applicabile |

Fare Acquisti:

| 1 | Fa tutte le proprie spese senza aiuto |
| --- | --- |
| 0 | Fa piccoli acquisti senza aiuto |
| 0 | Ha bisogno di essere accompagnato |
| 0 | Completamente incapace di fare acquisti |
| NA | Non applicabile |

Preparazione del cibo:

| 1 | Organizza, prepara e serve pasti adeguatamente preparati |
| --- | --- |
| 0 | Prepara pasti adeguati solo se sono procurati gli ingredienti |
| 0 | Scalda o serve pasti preparati oppure prepara cibi ma non mantiene una dieta adeguata |
| 0 | Ha bisogno di avere cibi preparati e serviti |
| NA | Non applicabile |

Governo della casa:

| 1 | Mantiene la casa da solo o con occasionale assistenza (per esempio aiuto per i lavori pesanti) |
| --- | --- |
| 1 | Esegue compiti quotidiani leggeri ma non mantiene un accettabile livello di pulizia della casa |
| 1 | Ha bisogno di aiuto in ogni operazione di governo della casa |
| 0 | Non partecipa a nessuna operazione di governo della casa |
| NA | Non applicabile |

Biancheria:

| 1 | Fa il bucato personalmente e completamente |
| --- | --- |
| 1 | Lava le piccole cose (calze, fazzoletti) |
| 0 | Tutta la biancheria deve essere lavata da altri |
| NA | Non applicabile |

Mezzi di trasporto:

| 1 | 1 Si sposta da solo sui mezzi pubblici o guida la propria auto |
| --- | --- |
| 1 | Si sposta in taxi ma non usa mezzi di trasporto pubblici |
| 1 | Usa i mezzi di trasporto se assistito o accompagnato |
| 0 | Può spostarsi solo con taxi o auto e con assistenza |
| 0 | Non si sposta per niente |
| NA | Non applicabile |

Responsabilità nell'uso dei farmaci:

| 1 | Prende le medicine che gli sono state prescritte |
| --- | --- |
| 0 | Prende le medicine se sono preparate in anticipo e in dosi separate |
| 0 | Non è in grado di prendere le medicine da solo |
| NA | Non applicabile |

Capacita' di maneggiare il denaro:

| 1 | Maneggia le proprie finanze in modo indipendente |
| --- | --- |
| 1 | E' in grado di fare piccoli acquisti |
| 0 | E' incapace di maneggiare i soldi |
| NA | Non applicabile |
